# Mobile Food Tracking Apps: Do They Provoke Disordered Eating Behavior? Results of a Longitudinal Study

**DOI:** 10.11621/pir.2024.0104

**Published:** 2024-03-01

**Authors:** Margarita S. Aslanova, Amina S. Valieva, Nataliya V. Bogacheva, Anastasia M. Skupova

**Affiliations:** a *I.M. Sechenov First Moscow State Medical University (Sechenov University), Russia*; b Federal Scientific Center of Psychological and Multidisciplinary Research, Moscow, Russia

**Keywords:** food tracking, food-tracking apps, disordered eating behavior, restrictive eating behavior, longitudinal study

## Abstract

**Background:**

Some studies connect the popularity of food-tracking apps to an increase in restrictive eating and other disordered eating behaviors and find those apps harmful for psychological well-being, but there is a lack of empirical studies, especially of Russian samples.

**Objective:**

To examine the connection between disordered eating symptoms, psychological well-being, and the use of a mobile food-tracking application.

**Design:**

The participants were 26 women aged 18–30 (*M* = 21.96; *SD* = 3.33); 24 completed the study. During the pre-test, the participants completed the Dutch Eating Behavior Questionnaire (Van Strien et al., 1985), the Eating Attitude Test (Skugarevskii, 2007), the Hospital Anxiety and Depression Scale (Ma et al., 2023), the Situation Inventory of Body-Image Dysphoria (Baranskaia & Tataurova, 2011), and a socio-demographic survey with additional questions related to food tracking, weight, and disordered eating. The experimental group was then tasked with tracking their food consumption with a mobile app for a month. The test battery was completed again immediately after the experiment ended, and for a third time one month later.

**Results:**

The comparative analysis showed a decrease in anxiety throughout the study, with a tendency-level increase in depressive symptoms by the end of the experiment. Contrary to expectations, emotional and external eating decreased during the experiment, while restrictive eating did not change. However, the risk of general disordered eating behavior increased one month after the experiment. The correlations between psychological well-being and eating behavior changed during the study. Immediately after the experiment, more correlations between eating behavior, body dissatisfaction, anxiety, and depression emerged, while at the later cutoff, correlations with depression and anxiety became insignificant.

**Conclusion:**

The study had mixed results, contradicting some previous research. Both emotional and external eating decreased along with anxiety levels; however, general disordered eating symptoms increased after food tracking.

## Introduction

### Eating Disorders

Eating behavior is a broad term that includes values-based attitudes towards food and its consumption, eating stereotypes in everyday life or stressful situations, as well as one`s own body image and body-image-related behaviors (Mendelevich, 2005). Normally, human eating behavior is balanced and includes various adaptive eating patterns; however, disordered eating behaviors (DEBs) also occur. The most common of these are: 1) *external eating,* characterized by high sensitivity towards external food-related stimuli, such as food appearance, smell, or advertisement, rather than internal ones, such as blood glucose level, or an empty stomach; 2) *emotional eating,* characterized by food consumption due to emotional discomfort rather than physical hunger; 3) *restrictive eating,* characterized by excessive food restriction and inconsistent dieting due to the desire to achieve weight loss or to prevent weight gain (Malkina-Pykh, 2022).

Eating disorders (diagnosed behavioral disorders that involve abnormal eating behaviors that are explained by psychological conditions [ICD-11, 2023]) can be described as clinically significant cases of DEBs. Eating disorders (EDs) include the following: 1) anorexia nervosa; 2) bulimia nervosa; 3) binge eating disorder; 4) avoid-ant-restrictive food intake disorder; 5) pica; 6) rumination-regurgitation disorder; and 7–8) other specified and unspecified feeding or eating disorders.

Non-clinical DEBs are not classified as psychiatric disorders because of their less evident symptoms, which are not prominent enough to fit the diagnostic requirements. Symptoms of DEBs are less intense, do not impact health significantly compared to EDs, and, unlike EDs, can appear temporarily.

Both DEBs and EDs are more common in women, suggesting that they are at higher risk of developing them (Arija et al., 2022; Erskine & Whiteford, 2018; Smink et al., 2012; Udo & Grilo, 2018). The prevalence of EDs in the world among women is 2.58% compared to .74% among men, confirming the higher susceptibility of the former to these types of disorders (Qian et al., 2021). One possible explanation is that a difference in psychological well-being is the cause: positive self-acceptance is less typical for women (Carter et al., 2013; Matud et al., 2019).

### Food-Tracking Mobile Applications

Modern worldwide trends and social influences have an impact on human eating behavior (Higgs & Ruddock, 2020; Pike et al., 2014). For example, food tracking became popular due to technological advances, such as the emergence of special mobile applications (apps) (Franco et al., 2016). There is also a trend towards an increase in screen time and the use of mobile applications among children (Cerniglia & Cimino, 2020; Ribner et al., 2021). Apps are simple and convenient to use. They include food-and calorie-tracking functions, estimate the amounts of proteins, fats, and carbohydrates in the daily diet, and, to an extent, track physical activity level. Most importantly for some users, food-tracking apps allow them to set and achieve weight loss goals more effciently through self-control (Aguilar et al., 2014; Burke et al., 2011; Cavero-Redondo et al., 2023; Gilmore et al., 2014). However, food-tracking apps are also among the factors that predict DEB progression (Hahn & Hazzard et al., 2022; Hahn & Linxwiler et al., 2021; Hahn & Sonneville et al., 2021; Levinson et al., 2017; Messer et al., 2021; Simpson et al., 2017).

Scientific interest in mobile food tracking apps has been increasing since 2017. Eating behavior researchers suggest that such applications may:

- have a negative impact on the psychological well-being of users (Hahn & Linxwiler et al., 2021; Levinson et al., 2017; Messer et al., 2021; Simpson et al., 2017);
- contribute to the development and/or maintenance and/or aggravation of restricting eating behavior (Hahn & Hazzard et al., 2022; Hahn & Linxwiler et al., 2021; Hahn & Sonneville et al., 2021; Levinson et al., 2017; Messer et al., 2021; Simpson et al., 2017);
- sustain excessive physical activity that leads to inanition and/or physical trauma (Hahn & Hazzard et al., 2022);
- contribute to the uncontrolled use of dietary supplements for weight loss or for the development of muscle mass (Hahn & Sonneville et al., 2021).

Currently there is a lack of empirical research on the correlations between food-tracking apps and DEB symptoms, despite the growing interest in the topic among scientists. The existing studies emphasize the need for additional validation of the results obtained from limited survey samples (Hahn & Hazzard et al., 2022; Hahn & Linxwiler et al., 2021; Hahn & Sonneville et al., 2021; Levinson et al., 2017; Messer et al., 2021; Simpson et al., 2017). No studies on the correlation between food-control apps and DEB symptoms have ever been conducted on a Russian sample, to our knowledge.

The purpose of this study is to explore DEB symptoms in women using FatSecret, a popular mobile food-tracking application. The main hypothesis of the study is that food-tracking app use has a negative impact on psychological well-being and can lead to DEB symptoms, specifically restrictive eating, among female users.

## Methods

### Instruments

An overview of mobile food-tracking apps in Russia was conducted prior to the experimental part of the study. At this stage, applications that were suitable for the most App Store and Google Play users (for example, Lifesum, YAZIO) were rated by several functionality-based criteria (*Table 1*). No significant differences were found among those apps; they have the same functionality. The decisive criterion of choice was the food-tracking application’s popularity among users. The FatSecret app was selected according to its average rating on the App Store and Google Play

**Table 1:** Comparison of popular food-tracking apps (FatSecret, Lifesum, and YAZIO)

	FatSecret	Lifesum	YAZIO
Accessibility (can be used both on Android and IOS, free version)	+	+	+
Calculation of calories, carbs, fats, and proteins	+	+	+
Ability to create individual recipes in the app	+	+	–
Hydration tracker	–	+	+
Physical-activity tracker	+	+	+
Synchronization with other apps	+	–	–
Average (App Store user and scores Google by platform Play)	4.8 and 4.6	4.7 and 4.4	4.8 and 4.5

The following diagnostic battery was used in the current study (for the exact procedures, periodicity, and number of measurements, see “Procedure”):

*The Dutch Eating Behavior Questionnaire (DEBQ)* was used to evaluate the severity of eating behavior styles: external eating (ExE), emotional eating (EmE), and restrained (restrictive) eating (ReE) (Van Strien et al., 1985). The questionnaire consists of 33 questions: 10 on the ExE and ReE scales and 13 on the EmE scale. The minimum score for restrictive and external eating scales is 10, and the maximum is 50. On the emotional eating scale, the minimum score is 13, and the maximum score is 65 points. The average scores on the ExE, EmE, and ReE subscales for people with weights within the normal range are 2.7, 1.8, and 2.4 points, respectively. If the score on one of the scales is higher than average, it might indicate the presence of disordered eating behavior patterns.

*The Eating Attitude Test (EAT-26)* is a screening self-report measure designed to identify the risk of disordered eating behavior (Skugarevskii, 2007). This questionnaire is used for a preliminary evaluation of a person`s attitudes towards eating. It consists of 26 statements, with the minimum cumulative score being 0 and the maximum score 78. If the cumulative score is more than 20, there is a probability of DEB.

*The Hospital Anxiety and Depression Scale (HADS)* is used in general medical practice for screening anxiety and depression (Ma et al., 2023). The scale consists of 14 statements: 7 relate to anxiety and 7 to depression, forming two subscales. The minimum score for each subscale is 0, and the maximum score is 21. The cutoffs for each subscale are: 0–7 (normal score; no significant symptoms of anxiety or depression are detected); 8–10 (subclinical levels of anxiety or depression); 11 and more (clinically significant anxiety and/or depression).

*Situation Inventory of Body-Image Dysphoria (SIBID)* is used to assess negative body image affect under different circumstances (Baranskaia & Tataurova, 2011). The questionnaire consists of 20 statements, with a minimum score of 0 and a maximum score of 80. All scores are summed with no subscales. For cutoffs, the scores of the participants (N = 114) were divided into quartiles; the cumulative score of 49 or higher was the cutoff for significantly negative body image affect.

The original survey form was designed to collect social and demographic data and information about prior experience with any food-tracking apps. The form included questions about the participants’ biological sex, age, height, and weight. It also included questions aimed at detecting previously diagnosed eating disorders and experience with food-tracking apps during the last six months. For example, “What is your weight in kg?”; “Have you ever been diagnosed with an eating disorder?”; “Have you been using any food self-control tools (to count calories) during the last 6 months?”; “Rate on a scale from 1 to 10: how much did the food-tracking app worsen your psychological well-being?”

### Participants

**Figure 1.**
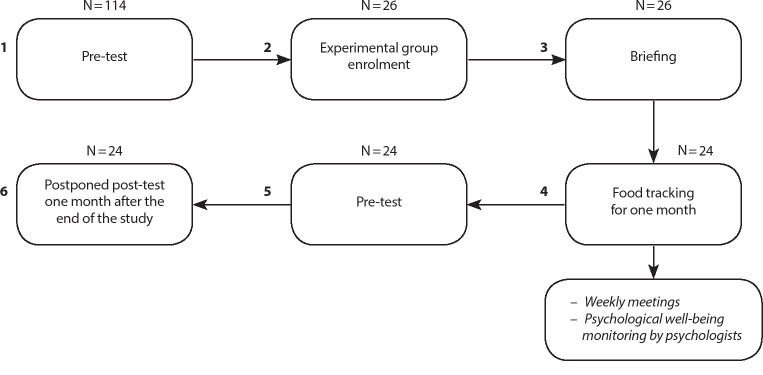
Stages of the study

The primary sample comprised 114 people aged 15–38 (*M* = 20.91; *SD* = 3.57). All respondents lived in Russia. They received a link with the questionnaires on the Internet. Later, 26 women, who gave consent, were selected from the sample to participate in the experiment. Then, during the experiment, two women claimed their psychological well-being deteriorated significantly and were immediately withdrawn from the study before its end (Figure 1).

Inclusion criteria for the experimental group were: 1) being female; 2) age above 18; 3) no prior experience with any kind of food-tracking forms or apps during the last 6 months; and 4) no diagnosed eating disorder. The final sample participants were aged 18–30 (*M* = 21.96; *SD* = 3.33) with BMI between 16.82–30.45 (*M* = 21.33; *SD* = 3.67), thus ranging from underweight (moderate thinness) to obese (class I).

### Procedure

The study was conducted from November 2022 to January 2023. It consisted of the following stages: 1) Pre-test: the participants completed the forms to be enrolled in the experimental group; 2) Briefing: A briefing session with the participants where they were told about the study, its goals and procedures; the participant’s rights were discussed, including voluntary participation, informed consent, and the possibility to leave the study at any time; 3) Experimental part: the participants controlled their food intake for one month using the FatSecret app; 4) Post-test: the participants completed the forms right after they finished their one-month food-tracking experience; 5) Postponed post-test: the participants completed the forms one month after they stopped food tracking (Figure 1). The questionnaire data was collected online via Google Forms.

All the participants were instructed to share their weekly statistics of food intake during the whole period of using the application. Each participant was instructed on how to create and use their FatSecret accounts. For the weight goal, the participants were instructed to maintain their current weight without attempting to alter it during the study. Weekly group meetings with the research supervisors were organized in free format for the participants to discuss their current psychological well-being. In case of emergencies, the participants were provided with psychological help hot-line contacts. After the first two weeks of the experiment, two women claimed their psychological well-being had deteriorated and ended their participation in the research.

## Results

### Participants’ Psychological Well-Being and Eating Behavior at Different Stages of the Study: Comparative Analysis

The comparison of women’s psychological well-being scores at the different stages of the study was made with Friedman’s chi-square test. There were significant differences in anxiety levels (chi-square = 8.667, *p* = .013) during all stages of the research (*Table 2*). The mean value analysis showed significant anxiety reduction during the whole time (pre-test > post-test > postponed post-test; the mean value difference was less than 1 point). There were no significant differences in depression intensity or body image dissatisfaction (*Table 2*).

**Table 2:** Differences in psychological well-being and eating behavior through the stages, with statistical significance levels (Friedman’s chi-square test)

	Statistical significance (Friedman’s chi-square test, significance level)	Mean, standard deviation	
	***Psychological well-being***		
Anxiety	χ^2^= 8.6X *p* = .013	Pre-test Post-test Postponed post-test	*M* = 8.67 *SD* = 3.39 *M* = 8.42 *SD* = 4.33 *M* = 7.5 *SD* = 3.58
Depression	χ^2^ =2.909 *p* = .234	Pre-test Post-test Postponed post-test	*M* = 4.54 *SD* = 3.42 *M* = 5.58 *SD* = 3.47 *M* = 4.33 *SD* = 2.82
Body dissatisfaction	χ^2^= 1.326 *p* = .515	Pre-test Post-test Postponed post-test	*M* = 28.04 *SD* = 15.47 *M* = 26.96 *SD* = 18.65 *M* = 27.46 *SD* = 27.46
	***Eating behavior***		
ExE	χ^2^ = 26.289 *p*< .001	Pre-test Post-test Postponed post-test	*M* = 3.24 *SD* = .73 *M* = 2.85 *SD* = .71 *M* = 3.15 *SD* = .79
EmE	χ^2^= 7.457 *p* = .024	Pre-test Post-test Postponed post-test	*M* = 2.83 *SD* = 1.04 *M* = 2.63 *SD* = 1.02 *M* = 2.56 *SD* = .89
ReE	χ^2^= 1.209 *p* = .546	Pre-test Post-test Postponed post-test	*M* = 2.43 *SD* = 1.04 *M* = 2.42 *SD* = .97 *M* = 2.40 *SD* = .90
EAT-26	χ^2^= 3.881 *p* = .144	Pre-test Post-test Postponed post-test	*M* = 6.08 *SD* = 6.02 *M* = 6.54 *SD* = 6.03 *M* = 7.83 *SD* = 6.39

The comparison of women’s eating behavior during different stages of the study with Friedman’s chi-square test showed significant differences in the levels of emotional eating (chi-square = 7.457, *p* = .024) and external eating (chi-square = 26.289, *p* < .001) (*Table 2*). Statistically significant differences in the severity of restrictive eating behavior and disordered eating behavior risk were not found (*Table 2*).

Pair-wise comparison of women’s psychological well-being and eating behavior styles before food tracking (1) and right after its end (2) with Wilcoxon signed-rank test showed that:

- there were tendency-level differences in depression symptoms (*T* = –1.692, *p* = .091) (Table 3). The mean value analysis showed an increase in depression symptoms right after the food tracking ended (pre-test < post-test; the mean value difference was more than one point). Depression symptom scores almost returned to their initial level one month after the food tracking ended (post-test > postponed post-test);
- there were differences in intensity of external (*T* = –4.097, *p* **<** .001) and emotional eating (*T* = –1.801, *p* = .072) (*Table 3*). The mean value analysis showed a decrease in intensity of ExE and EmE right after the food tracking ended (pre-test > post-test; the mean value difference was less than one point).

**Table 3:** Pair-wise comparisons of psychological well-being and eating behavior scores before the food tracking, right after it stopped, and one month later (Wilcoxon signed-rank test)

		Wilcoxon signed–rank test		
	Pair-wise comparison	*T*–score	Asymptomatic value	Differences
	*Psychological well-being*			
Anxiety	Pre-test — post-test Post-test — postponed post-test Pre-test — postponed post-test	–.889 –2.705 –1.237		Post-test > postponed post-test
Depression	Pre-test — post-test Post-test — postponed post-test Pre-test — postponed post-test	–1.692 –.609 –1.380		Pre-test < post-test
	*Psychological well-being*			
Body dissatisfaction	Pre-test — post-test Post-test — postponed post-test Pre-test — postponed post-test	–.844 –.335 –.261		
	*Eating behavior*			
ExE	Pre-test — post-test Post-test — postponed post-test Pre-test — postponed post-test	–4.097 –1.568 –3.409	<.001 .117 .001	Pre-test > post-test Pre-test < postponed post-test
EmE	Pre-test — post-test Post-test — postponed post-test Pre-testpost- — postponed test	–1.801 –2.132 –.697	.072 .033 .486	Pre-test > post-test Post-test > postponed post-test
ReE	Pre-test — post-test Post-test — postponed post-test Pre-test — postponed post-test	–.247 –.523 –.162	.805 .601 .872	
EAT-26	Pre-test — post-test Post-test — postponed post-test Pre-test — postponed post-test	–.053 –1.615 –1.745	.958 .106 .081	Pre-test < postponed post-test

At this stage, there were no statistically significant differences found in anxiety, body image dissatisfaction, restrained eating, and disordered eating behavior risk scores (*Table 3*).

Pair-wise comparison of women’s psychological well-being and eating behavior styles right after finishing the food tracking (2) and one month later (3) with Wilcoxon signed-rank test showed that:

- there were differences in the disordered eating behavior risk scores (*T* = –1.745, *p* = .081) (Table 3). The mean value analysis showed an increase in the disordered eating behavior risk scores one month after the food tracking ended (post-test < postponed post-test; the mean value difference was more than one point).
- there were significant differences in external eating (*T* = –3.409, *p* **=** .001) (*Table 3*). The mean values analysis showed an increase in ExE scores one month after the food tracking ended (post-test < postponed post-test; the mean value difference was less than one point).

At this stage, a pair-wise comparison of women’s psychological well-being scores right after the food tracking ended and one month later showed no statistically significant differences. There were also no significant differences in restrictive and emotional eating (Table 3).

Pair-wise comparison of women’s psychological well-being scores and eating behavior styles before the food tracking (1) and one month after the food tracking ended (3) with Wilcoxon signed-rank test showed that:

- there were significant differences in anxiety levels (*T* = –2.705, *p* = .007) (Table 3). The mean values analysis showed a decrease in anxiety intensity one month after the food tracking ended (pre-test < postponed post-test; the mean value difference was less than one point).
- there were significant differences in intensity of emotional eating (*T* = –2.132, *p* = .033) (Table 3). The mean values analysis showed a decrease in EmE scores one month after the food tracking ended (pre-test < postponed post-test; the mean value difference was less than one point).

At this stage there were no statistically significant differences in intensity of external and restrictive eating, disordered eating behavior risk scores, depression levels, and body dissatisfaction (*Table 3*).

### Correlational Analysis of Women’s Psychological Well-Being and Eating Behavior

At the next stage of statistical analysis of the collected data (using Spearman’s rank correlation coeffcient), we evaluated the correlations between women’s psychological well-being characteristics and eating behavior during different stages of the study (before the food tracking (1), right after the food tracking ended (2), and one month after the food tracking ended (3)).

**Figure 2.**
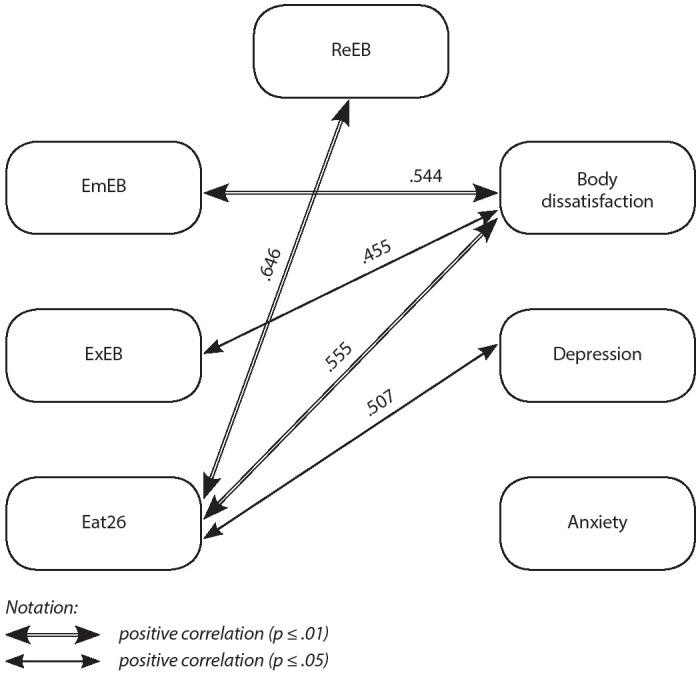
Correlations between women’s psychological well-being and eating behavior prior to the food-tracking experiment

The analysis showed several correlations between women’s psychological wellbeing characteristics and eating behavior prior to food tracking (*Figure 2*):

- there was a positive correlation between restrictive eating behavior and disordered eating behavior risk (*r* = .646, *p* = .001);
- there was a positive correlation between emotional eating and body dissatisfaction (*r* = .544, *p* = .006);
- there was a positive correlation between external eating and body dissatisfaction (*r* = .455, *p* = .025);
- there was a positive correlation between disordered eating behavior risk and depression symptoms severity (*r* = .507, *p* = .012) and body dissatisfaction (*r* = .555, *p* = .005).

Statistically significant correlations with anxiety were not found at this time cutoff.

**Figure 3.**
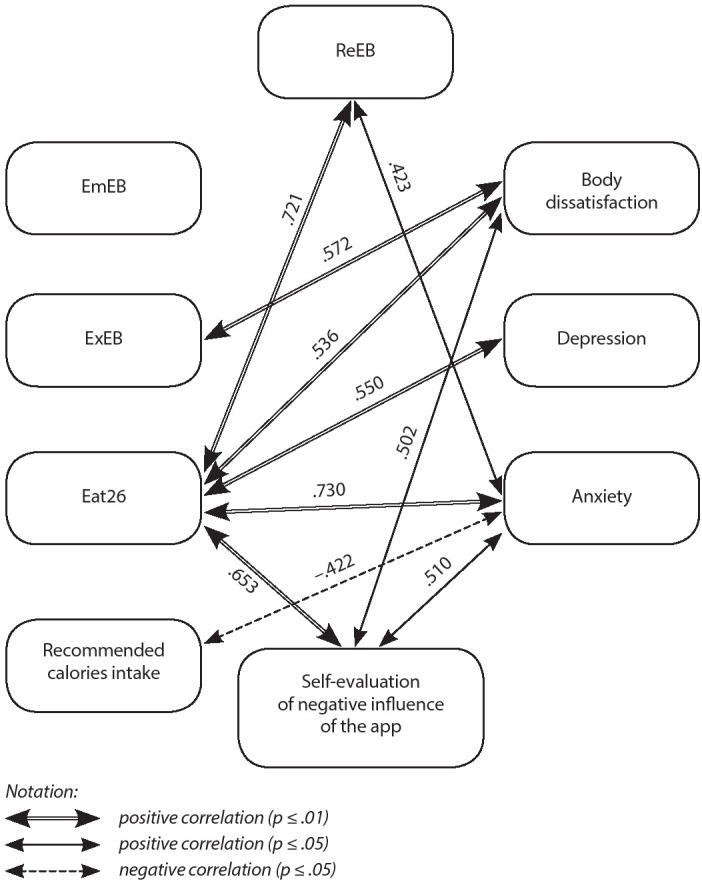
Correlation characteristics right after finishing the food tracking

Several correlations were found between women’s psychological well-being, eating behavior, and calorie intake as recommended by the food-tracker app at the cutoff point immediately after the food-tracking experiment was finished (*Figure 3*):

- the risk of disordered eating behavior was positively correlated with restrained eating scores (*r* = .721, *p* < .001), body dissatisfaction (*r* = .572, *p* < .007), depression (*r* = .550, *p* < .005), anxiety (*r* = .730, *p* < .001), and the self-evaluated negative influence of the food-tracking app on the participant’s psychological well-being (*r* = .653, *p* = .001);
- anxiety levels were positively correlated with restrictive eating (*r* = .423, *p* = .04) and the self-evaluated negative influence of the food-tracking app on the participant’s psychological well-being (*r* = .510, *p* = .011), and negatively correlated with the recommended calorie intake (*r* = –.422, *p* = .04);
- body dissatisfaction was positively correlated with the self-evaluated negative influence of the food-tracking app on the participant’s psychological wellbeing (*r* = .502, *p* = .012) and external eating intensity (*r* = .572, *p* = .003);

Statistically significant correlations with emotional eating were not found at this time cutoff.

**Figure 4.**
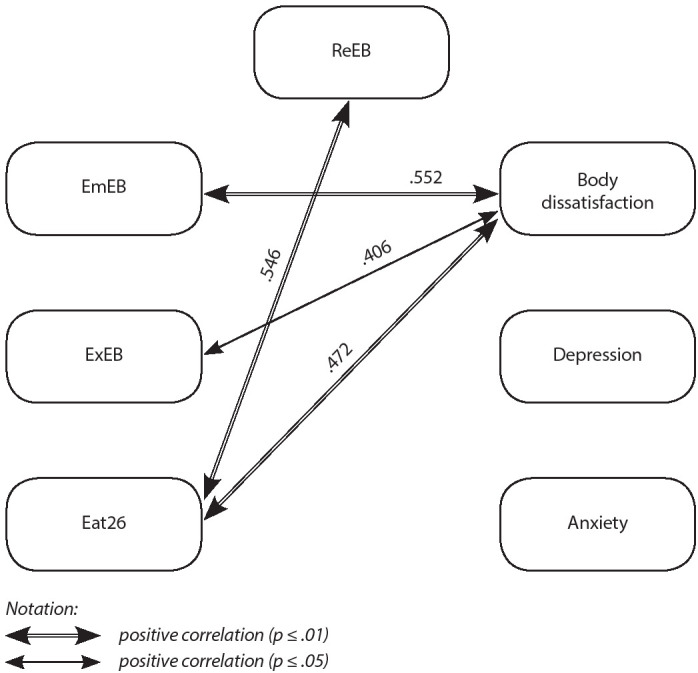
Correlations between women’s psychological well-being and eating behavior one month after the food-tracking experiment ended

Significant correlations were found between women’s psychological well-being and eating behavior one month after the food-tracking experiment ended (*Figure 4*):

- restrictive eating behavior was positively correlated with disordered eating behavior risk (*r* = .546, *p* = .006);
- body dissatisfaction was positively correlated with emotional (*r* = .552, *p* = .005) and external eating (*r* = .456, *p* = .025) and the risk of disordered eating behavior (*r* = .472, *p* = .02).

Statistically significant correlations between anxiety and depression were not found at this cutoff point.

Therefore, established correlations changed at different points of the study compared to the pre-test results. **Right after the food tracking experiment ended, the following correlations were found:**

- between anxiety and restrictive eating (*r* = .423, *p* = .04) and recommended calorie intake (*r* = –.422, *p* = .04);
- between self-evaluated negative influence of the app on participant’s psychological well-being and the risk of disordered eating (*r* = .653, *p* = .001), anxiety (*r* = .510, *p* = .011), and body dissatisfaction (*r* = .502, *p* = .012).
- The correlation between emotional eating and body dissatisfaction disappeared at this time cutoff (*r* = .402, *p* = .051).

Then, the following correlation changed at the last time cutoff of the study, **one month after the food tracking ended** (compared to the “before food tracking” cutoff): between depression and the risk of disordered eating behavior (*r* = .17, *p* = .427).

To generalize, correlations between the psychological well-being and eating behavior have been changing throughout the study.

## Discussion

This study aimed to explore disordered eating symptoms and psychological wellbeing among women who use food-tracking apps. We found that the general level of DEB risk increased closer to the end of the study; however, certain DEBs (emotional and external eating) decreased. This could be due to the “display of calories consumed” function of the application. Presumably, when the participants opened the application and saw the number of calories consumed, they experienced emotional discomfort. But no differences were found in the severity of restrictive eating behavior, which contradicts the previous studies on the use of food-tracking apps and the development of DEB symptoms (Hahn & Hazzard et al., 2022; Hahn & Linxwiler et al., 2021; Hahn & Sonneville et al., 2021; Levinson et al., 2017; Messer et al., 2021; Simpson et al., 2017). The authors of those studies concluded that regular calorie counting was connected to anxiety after meals and an increased severity of restrictive eating. Such contradictions could be explained by the small size of our experimental group and some liberties taken with the methods. Specifically, the Dutch Eating Behavior Questionnaire (DEBQ) was used in its Russian version but had never been properly validated on a Russian sample, despite this questionnaire being commonly used in Russian studies.

Also, the severity of the depressive symptoms increased in the participants right after the end of the food tracking. That data did not accord with the results found by Hahn, Kaciroti, et al. (2021), where there was no correlation between depression and nutrition control. However, unlike in the abovementioned research, there was also an increased general level of DEB risk among the participants in the current study. That parameter could have influenced the severity of depression symptoms and led to their increase. This is coherent with the other studies in the field, where a connection between DEB symptoms and depression was shown (Garcia et al., 2020; Martín et al., 2019; Mason & Lewis, 2014).

Moreover, there was a decrease in the general level of anxiety found during the whole study. However, Hahn, Kaciroti, et al. (2021) found no correlations between food tracking and anxiety, which does not cohere with the results of our study. Our assumption is that a decrease in the general level of anxiety appeared because the participants obtained a tool (the food-tracking application) to deal with their anxiety, with the sense of being in control of their food consumption. This feeling of control over one’s own behavior and eating habits could influence the general level of anxiety. Also, the app’s news feed, where users post their thoughts, could provide emotional support and reduce anxiety. The study also showed that introducing recommended calorie intake information may lead to an increase in anxiety. The anxiety scores among the participants increased when the application suggested more restrictions on food intake (by setting the recommended number of calories). Prior studies showed that there are indeed connections between restrictive eating behavior and anxiety (Schaumberg et al., 2021; Swinbourne & Hunt et al., 2012; Swinbourne & Touyz, 2007).

Our results showed changing correlations between psychological well-being and eating behavior among the participants before the food-tracking experiment and at its different stages. It is fair to assume that using the food-tracking app influenced the eating behavior of the participants. Particularly, the women’s anxiety, which was not connected with eating behavior prior to the study, emerged in the symptoms of restrictive eating (as anxiety increases, restrictive eating behavior becomes more prominent). Numerous studies confirm this correlation (Schaumberg et al., 2021; Swinbourne & Hunt et al., 2012; Swinbourne & Touyz, 2007).

## Conclusion

The study explored the correlations between the use of a food-tracking app and disordered eating behavior. The results suggest that during use of the food-tracking app, women’s psychological well-being changes: depressive symptoms increase while anxiety decreases. During the experiment, the connections between eating behavior and psychological well-being also changed. Our assumption is that pre-existing anxiety, unrelated to eating behavior, manifested itself in the form of restrictive eating behavior. However, no expected changes were found in restrictive eating behavior during use of the food-tracking application. Emotional and external eating decreased during the study, while the general risk of ED symptoms increased. The results were somewhat mixed and thus require further studies with larger samples and proper control for limitations.

Despite the topicality of the study, there is a lack of research in the area, both in Russia and worldwide. The findings of this study may become a base for further research on a Russian sample and may contribute to the development of a new food tracker or the updating of existing applications, accounting for their influence on users’ psychological well-being and eating behavior.

## Limitations

Several limitations must be taken into consideration while interpreting the results of the study: the small size of the experimental group (N = 26) and the specific methods used. In particular, the Dutch Eating Behavior Questionnaire (DEBQ) and the Situation Inventory of Body-Image Dysphoria (SIBID) were not validated on a Russian sample, even though their direct translations are widely used in Russian studies. The psychotherapeutic effects of the weekly meeting and the inability to fully control the regularity of the food tracking (e.g., the participants could delay their notes for a day after actually eating a meal) should also be taken into consideration.
